# Endoscopic Endonasal Transturbinate Approach to the Pterygopalatine Fossa in the Management of Juvenile Nasopharyngeal Angiofibromas

**DOI:** 10.1155/2012/786262

**Published:** 2012-06-26

**Authors:** Satoru Kodama, Hideaki Mabuchi, Masashi Suzuki

**Affiliations:** Department of Otolaryngology, Faculty of Medicine, Oita University, 1-1 Idaigaoka, Hazama-cho, Oita Yufu 879-5593, Japan

## Abstract

Pterygopalatine fossa (PPF) is a difficult-to-access anatomic area located behind the posterior wall of the maxillary sinus. Juvenile nasopharyngeal angiofibroma (JNA) often affects this area, and the management of feeding artery to the tumor is important in the surgery. Endoscopic endonasal approach to the PPF without endangering all other nasal structures is useful in the management of JNA. We describe a new approach to the PPF, endoscopic transturbinate approach, which is effective in the management of JNA. Submucous inferior turbinoplasty was performed, and sphenopalatine artery, the feeder to the tumor, was identified at the sphenopalatine foramen. The posterior wall of maxillary sinus was removed. Internal maxillary artery was identified in the PPF and was ligated with a hemoclip. The tumor in the PPF was pushed into the nasal cavity. These procedures were all performed via submucous turbinate tunnel. Then, the tumor was successfully removed in en bloc from the nasal cavity by transnasal approach without ethmoidectomy. This approach improves accessibility and visualization in the PPF and potential to reduce intraoperative bleeding due to ligation of the feeder safely without touching the tumor. Endoscopic transturbinate approach is effective in the management of early stage of JNA.

## 1. Introduction

 Pterygopalatine fossa (PPF) is a difficult-to-access anatomic area located behind the posterior wall of the maxillary sinus and is bordered by the pterygoid plates posteriorly and middle cranial fossa superiorly [1]. Juvenile nasopharyngeal angiofibroma (JNA) often affects this area, and the management of feeding artery to the tumor is important in the surgery [2]. We describe a new approach to the PPF, endoscopic transturbinate approach, which is effective in the surgical management of JNA. 

## 2. Case Presentation

**Figure 1:**
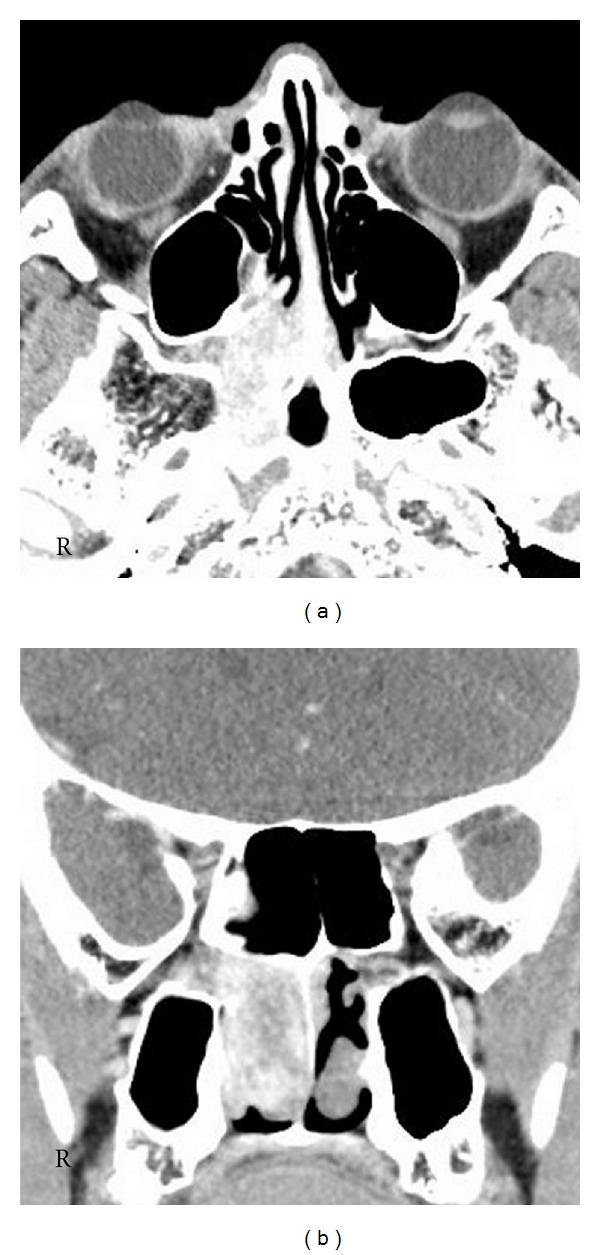
Enhanced axial (a) and horizontal (b) computed tomography (CT) images. Homogenous and strongly enhanced mass filling the posterior part of the right nasal cavity with extension to the pterygopalatine fossa (PPF) and sphenoid sinus.

 A 25-year-old man presented with recurrent right epistaxis. Nasal endoscopy revealed a red, easily bleeding mass that filled the posterior part of the right nasal cavity. Enhanced computed tomography (CT) showed strongly enhanced tumor in the right nasal cavity with the extension to the PPF. The tumor also extended to the sphenoid sinus with bone erosion (Figure 1). The tumor was considered as JNA, Radkowski stage IIA [3], without performing preoperative biopsy due to the risk of bleeding. The angiography showed that the feeder into the tumor was internal maxillary artery (IMA) and the sphenopalatine artery (SPA).

**Figure 2:**
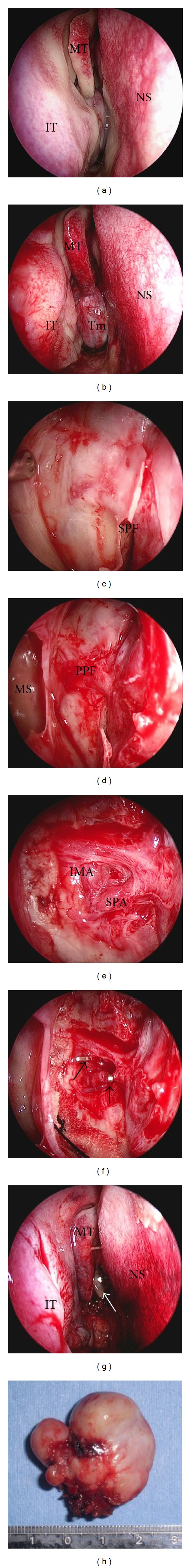
Preoperative (a), intraoperative (b), (c), (d), (e), and (f), postoperative endoscopic views (g), and macroscopic features of the excised tumor (h). (a) The tumor is filled in the posterior part of the right nasal cavity. (b) Submucous inferior turbinoplasty provides improved visualization and accessibility to the tumor. (c) Maxillary sinus is opened, and the mucosa is elevated from the posterior wall of the sinus. (d) The posterior wall bone of maxillary sinus is then removed, and the PPF is widely exposed endoscopically. (e) and (f) Internal maxillary artery and sphenopalatine artery are identified in the PPF and are ligated with a hemoclip. (g) and (h) The tumor is removed in en bloc from the nasal cavity. IT: inferior turbinate; MT: middle turbinate; NS: nasal septum; Tm: tumor; SPF: sphenopalatine foramen; PPF: pterygopalatine fossa; MS: maxillary sinus; IMA: internal maxillary artery; SPA: sphenopalatine artery. Black arrows: hemoclip, white arrows: sphenoid sinus.

 Endoscopic sinus surgery was performed under general anesthesia. Firstly, submucous inferior turbinoplasty (SIT) was performed. Vertical incision was made along the anterior margin of the inferior turbinate to expose the turbinate bone. The covering mucosa was elevated from the bone, and the inferior turbinate bone was removed with preserving the mucosa. Mucosal elevation was continued to expose the uncinate process. The inferior half of the uncinate process together with the horizontal portion of the inferior turbinate bone was then removed. SIT provided improved visualization and wide working space in the posterior part of the nasal cavity (Figures 2(a) and 2(b)). The inferior turbinate mucosa was preserved throughout the surgery without swinging the lateral wall mucosa into the nasal cavity. Mucosal elevation was then continued posteriorly, and SPA, the feeder to the tumor was identified at the sphenopalatine foramen. Maxillary sinus was opened, and the mucosa was elevated from the posterior wall of the sinus. The posterior wall bone of maxillary sinus was then removed, and the PPF was widely exposed endoscopically (Figures 2(c) and 2(d)). IMA was identified in the PPF and was ligated with a hemoclip, and IMA and SPA were cut with the Harmonic Scalpel (Figures 2(e) and 2(f)). The tumor in the PPF was pushed into the nasal cavity. These procedures were all performed via submucous turbinate tunnel and were able to archive to manage the feeding artery safely without touching the easily bleeding tumor. Then, the tumor was resected transnasally without ethmoidectomy and was successfully removed in en bloc from the nasal cavity (Figures 2(g) and 2(h)).

No nasal packing was needed, and the postoperative course was uneventful. Postoperative histologic examination confirmed the diagnosis of JNA. No recurrences were observed at the 2-year follow-up examination. The anatomical structure of the nasal cavity and the physiological function of the nose could be preserved by this surgical technique.

## 3. Discussion

 JNA is a benign tumor, but is locally aggressive and destructive disease. The extremely rich vascularization together with the complex anatomical structures on the cranium, including cranium nerves and vascular structures, and the aggressive growth pattern of the tumor makes its treatment a challenge. Surgical excision is the treatment of choice for JNA [3–5]. The tumor originates from the area of the sphenopalatine foramen and can spread through foramina and fissures. JNA frequently involves the PPF even in the early stage according to the Radkowski staging, and surgical management in the PPF is important in the management of JNA [2].

 PPF is a difficult-to-access anatomic area located behind the posterior wall of the maxillary sinus, and is bordered by the pterygoid plates posteriorly and the greater sphenoid wing and middle cranial fossa superiorly [1]. Conventional approaches to the PPF require transmaxillary techniques that violates the anterior and posterior walls of the maxillary sinus. Recently, endoscopic transnasal approach to PPF has been well studied and developed, and the safety and efficacy for resecting PPF tumors have been shown owing to improved accessibility and visualization [1, 6]. Recent anatomical study described three different endoscopic endonasal approaches to the PPF: (1) the endonasal middle meatal transpalatine approach, (2) the endonasal middle meatal transnasal approach, and (3) the endonasal inferior turbinectomy transnasal approach [6]. The first approach was suitable for medial exposure of the PPF contents and the second was useful to obtain a lateral view of the fossa. The third approach offered the widest view and room for surgical maneuvering in the medial and lateral compartments of the PPF [6]; however, middle turbinate and inferior turbinate are necessary to maintain the physiological turbulence of the nasal airstream. Endoscopic endonasal approach we have employed has been achieved without endangering all other nasal structure. SIT is frequently performed for the treatment of the hypertrophy of inferior turbinate [7]. SIT reduces the volume of the inferior turbinate, resulting in improved visualization and working space in the nasal cavity [8]. In addition, SIT does not decrease the physiological function of the nose with maintaining the anatomical nasal structure [7]. Thus, endoscopic transturbinate approach might be a minimally invasive approach to the PPF.

Although preoperative selective embolization of feeding arteries has decreased intraopreative blood loss and made it possible to resect even the large tumors, recently, endoscopic surgery for the early stage of JNA has been reported to be performed without preoperative embolization [9]. Endoscopic endonasal transturbinate approach to the PPF allowed the surgeon improved visualization and accessibility to the PPF. This enables to manage the feeder to the tumor safely and to reduce the intraoperative blood loss, leading to the reduced requirement of preoperative interventional radiology and less morbidity.

 In conclusion, endoscopic endonasal transturbinate approach to the PPF is effective for the management of the early stage of JNA.
